# Interatrial Communications: Prevalence and Subtypes in 12,385 Newborns–a Copenhagen Baby Heart Study

**DOI:** 10.1007/s00246-024-03571-0

**Published:** 2024-07-13

**Authors:** Elisabeth Blixenkrone-Møller, Sofie Dannesbo, Anna Maria Dehn, Christian A. Pihl, Anne-Sophie Sillesen, R. Ottilia B. Vøgg, Anna Axelsson Raja, Steven Colan, Luc Mertens, Niels Vejlstrup, Henning Bundgaard, Kasper Iversen

**Affiliations:** 1 Department of Cardiology, Copenhagen University Hospital Herlev, Borgmester Ib Juuls Vej 1, 2730 Copenhagen, Denmark; 2 Department of Clinical Medicine, University of Copenhagen, Copenhagen, Denmark; 3 Department of Cardiology, The Heart Centre, Copenhagen University Hospital Rigshospitalet, Copenhagen, Denmark; 4 Department of Cardiothoracic Surgery, The Heart Centre, Copenhagen University Hospital Rigshospitalet, Copenhagen, Denmark; 5 Department of Cardiology, Boston Children’s Hospital, Boston, USA; 6 Department of Cardiology, The Hospital for Sick Children, Toronto, Canada

**Keywords:** Interatrial communication, Patent foramen ovale, Atrial septal defect, Prevalence, Neonate, Echocardiography

## Abstract

The prevalence of interatrial communications in newborns, i.e., patent foramen ovale or atrial septal defect, was previously reported to be between 24 and 92%, but the area has been impeded by lack of a universal classification method. A recently published novel echocardiographic diagnostic algorithm for systematic classification of interatrial communications had inter-and intraobserver agreements superior to standard expert assessment. This study aimed to determine the prevalence of subtypes of interatrial communications on transthoracic echocardiography in newborns. Echocardiograms of newborns aged 0–30 days were prospectively collected in the population-based cohort study Copenhagen Baby Heart Study in 2017–2018 and analyzed according to the new diagnostic algorithm, classifying interatrial communications into three subtypes of patent foramen ovale and three subtypes of atrial septal defects. Echocardiograms from 15,801 newborns were analyzed; 3416 (21.6%) were excluded due to suboptimal image quality or severe structural heart disease (*n* = 3), leaving 12,385 newborns (aged 12 [interquartile range 8; 15] days, 48.2% female) included in the study. An interatrial communication was detected in 9766 (78.9%) newborns. According to the algorithm, 9029 (72.9%) had a patent foramen ovale, while 737 (6.0%) fulfilled criteria for an atrial septal defect, further divided into subtypes. An interatrial communication was seen on echocardiography in almost 80% of newborns aged 0–30 days. Patent foramen ovale was 12 times more frequent than atrial septal defects. The observed prevalence of atrial septal defects was higher than previously reported. Follow up studies could distinguish which interatrial communications require follow-up or intervention. ClinicalTrial.gov, NCT02753348, posted April 27, 2016, [https://classic.clinicaltrials.gov/ct2/show/NCT02753348].

**Supplementary Information:**

The online version contains supplementary material available at 10.1007/s00246-024-03571-0.

## Introduction

Interatrial communications (IACs) are seen on echocardiography in 24 to 92% of newborns [1–8]. These are either patency of the oval foramen (PFOs) or atrial septal defects (ASDs). While the vast majority of neonatal IACs will undergo spontaneous closure, approximately 25% of IACs persist throughout life [9, 10]. Although usually asymptomatic in childhood, IACs can lead to complications later in life such as cryptogenic stroke, right ventricular volume overload, and atrial fibrillation, and are associated with a higher long-term mortality [11–15]. Previous echocardiographic studies investigating the prevalence of IACs in newborns are limited by relatively small cohorts (*n* = 36–1389) and potential selection bias [1–5], either by only including newborns referred to pediatric care [3], infants with concomitant congenital heart disease that requires further examination and/or treatment [2], or by excluding infants born to mothers with chronic diseases such as diabetes mellitus and hypertensive disorders of pregnancy [4]. In previous studies of the prevalence of IAC, no differentiation between PFO and ASD was made [1, 4–7], or the differentiation was either not described [2] or was based solely on the size of IAC [3], making comparisons between the studies difficult.

**Fig. 1:**
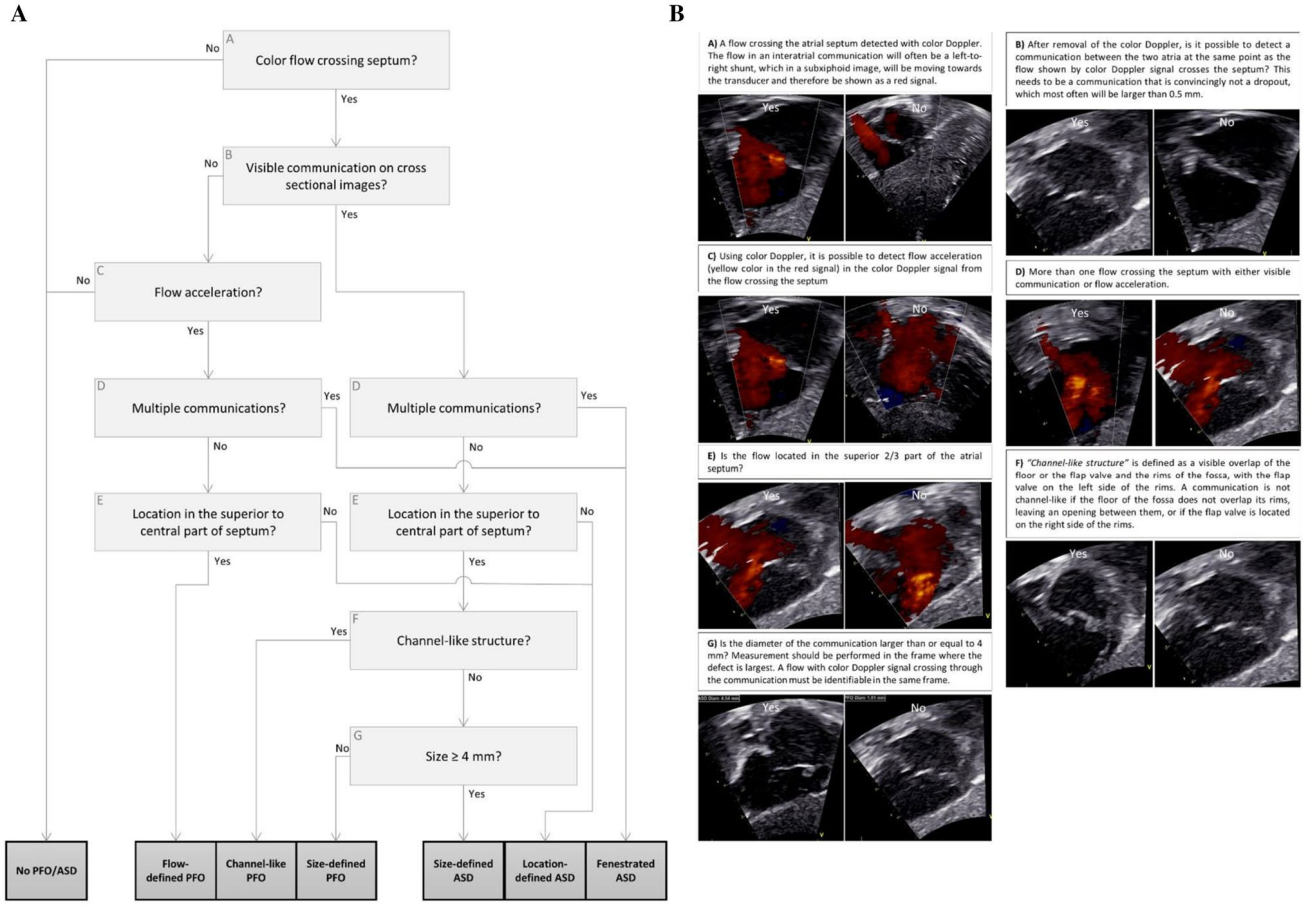
Algorithm for classification of interatrial communications. From Dannesbo, S. et al., 2022: *A novel algorithm for classification of interatrial communications within the oval fossa in the newborn—a Copenhagen Baby Heart Substudy. Cardio Young.* **A** Flowchart used in analyzing the atrial septum. **B** Description of flowchart. Material is used with permission

We have recently published a novel diagnostic algorithm for classifications of IACs [16] (Fig. 1 and Supplementary Figs. 1, 2).

The algorithm is based on transthoracic echocardiographic (TTE) findings in unselected newborns and takes the normal fetal development into account such as the formation and overlapping of the septum primum and septum secundum. The inter-and intraobserver agreement of the diagnostic algorithm was superior to standard assessment by experts.

The purpose of this study was to determine the prevalence and subtypes of IACs in the oval fossa by TTE based on this novel diagnostic algorithm in a large, unselected population of newborns.

## Methods

### Study Design and Cohort

This study is part of the Copenhagen Baby Heart Study, a prospective, multicenter, population-based cohort study focusing on cardiovascular health with inclusion of *n* = 27,595 newborns. The study design and cohort was previously described in details [17, 18]. In short, newborns born between April 2016 and October 2018 were included prenatally, and postnatal examination included a TTE within the first 2 months of life, preferably within 30 days after birth.

**Fig. 2:**
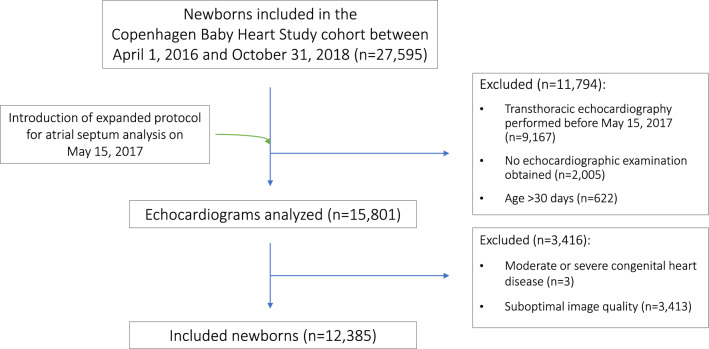
Consort diagram describing inclusion and exclusion of newborns in this cross-sectional study

The present study was cross-sectional and included newborns who had a TTE performed from May 15, 2017, as the echocardiographic protocol at this time was extended with images appropriate for analysis of the atrial septum according to the diagnostic algorithm. Echocardiograms of suboptimal imaging quality and of newborns aged > 30 days were excluded. Newborns with moderate or severe congenital heart disease according to Hoffman et al. [19] were excluded (Fig. 2).

### Echocardiographic Acquisition

**Fig. 3:**
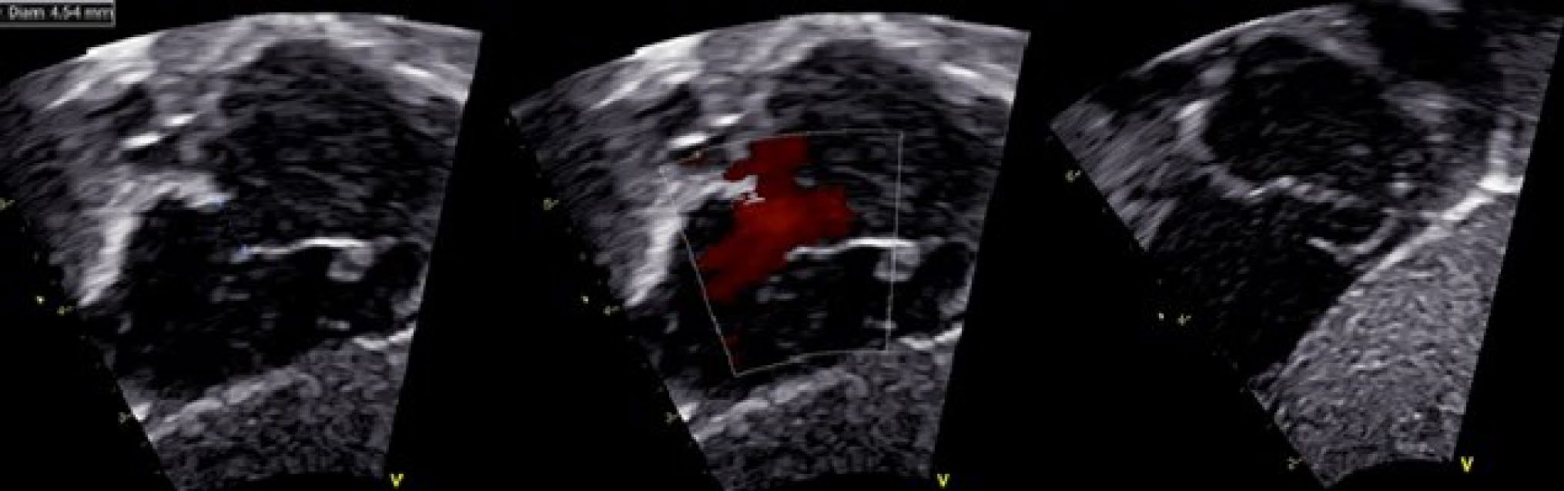
Three transthoracic echocardiographic images of the atrial septum in two subxiphoid views where the atrial septum is best visualized using cross-sectional images and color Doppler. A sweep through the atrial septum was performed to investigate the septum thoroughly and to capture a possible IAC

A standard pediatric TTE was performed using GE Vivid E9 (GE Vingmed Ultrasound, Horton, Norway) equipment by sonographers and physicians trained in pediatric echocardiography. The Copenhagen Baby Heart Study echocardiographic protocol has previously been described [17] and are in accordance with the American Society of Echocardiography’s guidelines for pediatric echocardiography [20]. Specific for assessment of the atrial septum, the protocol included cine loops with minimum three cardiac cycles obtained with a 6 mHz transducer from subxiphoid views; two images of the atrial septum in an oblique view in between a transverse and sagittal plane where the atrial septum was best visualized using cross-sectional images and color Doppler, and one image in a sagittal plane where the superior caval vein was visualized using color Doppler. A sweep through the atrial septum was performed to investigate the septum thoroughly (Fig. 3).

All images were stored digitally and analyzed offline using EchoPAC software v113 and v202 (GE Vingmed Ultrasound, Horton, Norway).

### Echocardiographic Analysis

Each image of the atrial septum was investigated in cine loop by one of two sonographers using the new algorithm [16] (Fig. 1 and Supplementary Figs. 1, 2) classifying IACs into 3 subtypes of PFO and 3 subtypes of ASD. The atrial septum was inspected frame-by-frame with color Doppler for identification of a flow crossing the septum and without color Doppler for identification of a visible communication in cross-sectional images. In the presence of an IAC, the size, location, number of IAC(s), and the morphology of the atrial septum were applied to the flowchart of the algorithm for classification of IAC subtype. The subtypes of PFO were as follows: flow-defined PFO visible on Doppler, channel-like PFO, and size-defined PFO with a diameter of < 4 mm. The subtypes of ASD were as follows: size-defined ASD with a diameter of ≥ 4 mm, fenestrated ASD with multiple communications in the atrial septum, and location-defined ASD with the location of the defect in the lower 1/3 part of the septum towards the atrioventricular valves.

### Ethics

The study was approved by the Regional Ethics Committee (H-16001518) and the Danish Data Protection Agency (I-Suite no.: 04546, ID-no. HGH-2016-53). Written informed parental consent was obtained prior to inclusion.

### Statistical Analysis

Categorical variables are compared using the Chi-square test. Continuous variables are presented as means with standard deviation (SD) and compared using ANOVA test when normally distributed, or else as medians and interquartile ranges [IQR] and compared using Kruskal–Wallis H test. Bonferroni correction for multiple testing was applied and *P* values < 0.005 were considered statistically significant (analyses for 11 demographic and echocardiographic parameters: *P* < 0.05/11 = 0.005). Statistical analyses were performed using R v4.2.2.

## Results

Echocardiograms of 15,801 newborns at the age of 0–30 days from the Copenhagen Baby Heart Study were available for this study. Three newborns were excluded due to moderate or severe structural heart disease, i.e., coarctation of the aorta (*n* = 1), severe valvular pulmonary stenosis (*n* = 1), and transposition of the great arteries (*n* = 1). 3413 (21.6%) newborns were excluded due to suboptimal image quality, leaving 12,385 newborns (median age 12 [IQR 8;15] days, 48.2% female) (Fig. 2). Demographic and echocardiographic characteristics of the newborns and maternal demographic characteristics are presented in Table 1.

**Table 1:** Characteristics for newborns with ASD, PFO, and no IAC visible on transthoracic echocardiography

Demographic and echocardiographic characteristics	No IAC (*n* = 2,619)	PFO (*n* = 9,029)	ASD (*n* = 737)	All (*n* = 12,385)	*P*-value^a^
Age in days, median [IQR]	10 [6;13]	12 [8;16]	10 [6;14]	12 [8;15]	P < 1·10^–4^
Gestational age, median, w + d [IQR in days]	40 + 1 [14]	40 + 1 [13]	40 + 0 [15]	40 + 1 [14]	*P* = .03
Sex, female, No. (%)	1335 (51.0)	4239 (46.9)	391 (53.1)	5965 (48.2)	*P* < 1·10^–4^
Twin, No. (%)	86 (3.3)	234 (2.6)	21 (2.8)	341 (2.8)	*P* = .16
Birth length, cm, mean (± SD)	51.5 (2.3)	51.6 (2.4)	51.3 (2.4)	51.6 (2.4)	*P* = 1·10^–4^
Birth weight, g, mean (± SD)	3471 (519)	3507 (524)	3466 (527)	3497 (523)	*P* = .002
Ventricular septal defect, No. (%)	75 (2.9)	294 (3.3)	41 (5.6)	410 (3.3)	*P* = .001
Bicuspid aortic valve, No. (%)	16 (0.6)	84 (0.9)	5 (0.7)	105 (0.8)	*P* = .26
Maternal characteristics					
Maternal age at delivery, y, mean (± SD)	31.7 (4.6)	31.7 (4.6)	31.8 (4.7)	31.7 (4.6)	*P* = .65
Maternal prepregnancy BMI, mean (± SD)	23.6 (4.3)	23.5 (4.4)	23.9 (4.6)	23.6 (4.4)	*P* = .08
Maternal smoking during pregnancy^b^, No. (%)	88 (3.4)	315 (3.5)	30 (4.1)	433 (3.5)	*P* = .65

*ASD* Atrial septal defect, *IAC* Interatrial communication, *IQR* Interquartile range, *PFO* Patent foramen ovale, *SD* Standard deviation

^a^Significance level was set at *P* < .05. When Bonferroni correction for multiple testing was applied, the significance level was set at *P* < .005; (0.05/11 = 0.005). With Bonferroni correction no significant difference in gestational age was observed between the groups of newborns

^b^Mothers answering “yes” or “stopped” when asked about smoking during pregnancy were placed in the same group, as those who stopped smoking during pregnancy might have been smoking during the 4th and 5th embryological week, where the atrial septum is being developed

### Prevalence of IACs and Subtypes

**Fig. 4:**
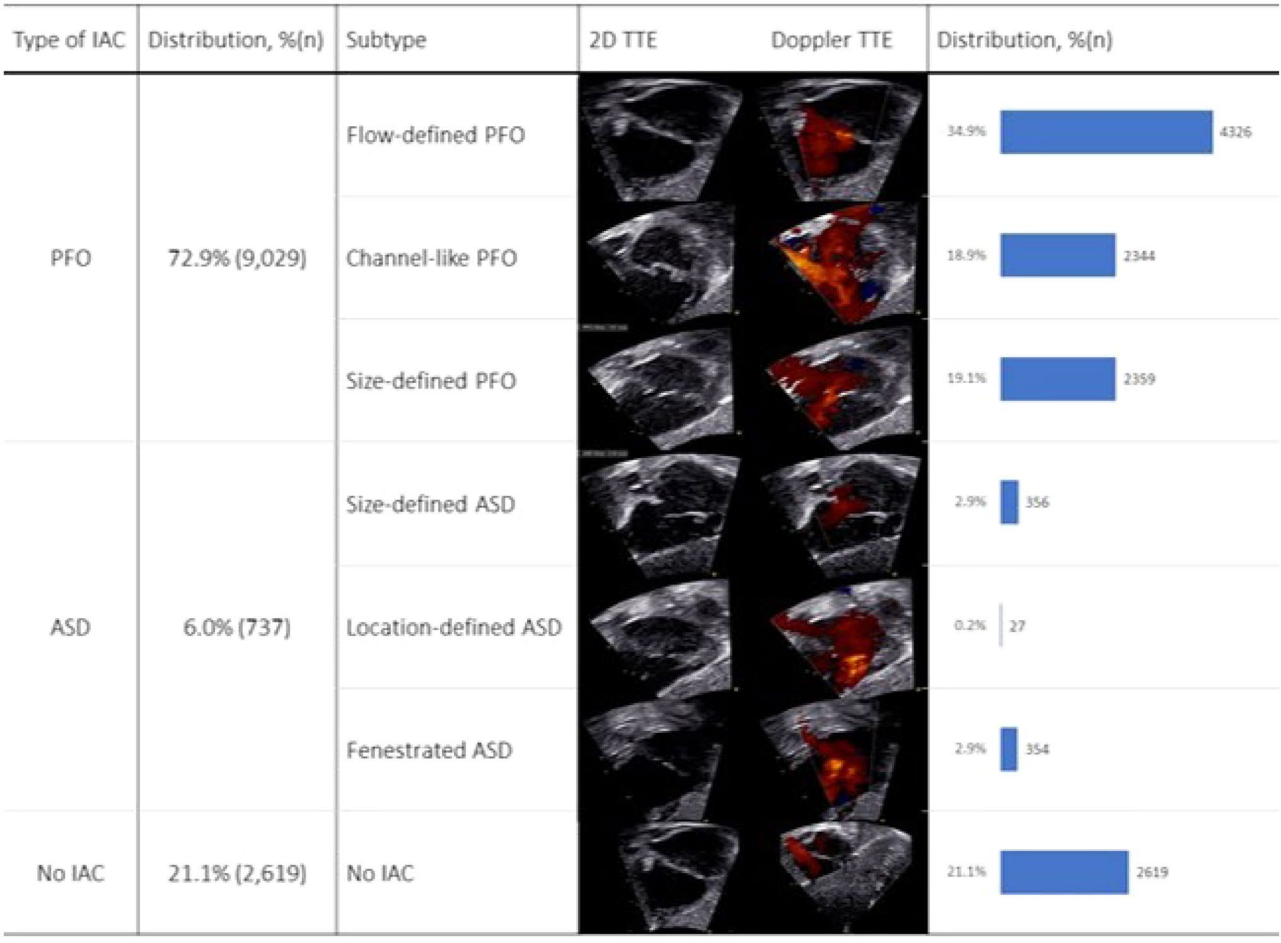
Distribution of subtypes of interatrial communications and transthoracic echocardiographic views *IAC* Interatrial communication. *ASD* Atrial septal defect, *PFO* Patent foramen ovale, *TTE* Transthoracic echocardiography, *2D* Cross-sectional view, *n* = 12,385

An IAC was visible in 9766 (78.9%) newborns, of which 9029 (72.9%) were classified as a PFO, while 737 (6.0%) fulfilled criteria for an ASD. The IACs were distributed into subtypes (Table 2 and Fig. 4).

**Table 2:** Prevalence of subtypes of IACs during the first four weeks of life

Subtype of IAC	Prevalence (No.)				
	First week	Second week	Third week	Fourth week^a^	Overall
Size-defined ASD	5.3% (128)	2.7% (159)	1.8% (50)	1.4% (19)	2.9% (356)
Location-defined ASD	0.2% (4)	0.3% (17)	0.2% (5)	0.1% (1)	0.2% (27)
Fenestrated ASD	2.6% (64)	2.6% (153)	3.1% (87)	3.7% (50)	2.9% (354)
Flow-defined PFO	27.6% (672)	35.6% (2,067)	37.5% (1,046)	39.6% (541)	34.9% (4,326)
Channel-like PFO	7.6% (185)	17.9% (1,036)	26.6% (740)	28.0% (383)	18.9% (2,344)
Size-defined PFO	27.7% (673)	18.9% (1,097)	15.8% (441)	10.8% (148)	19.1% (2,359)
No IAC	29.0% (704)	22.0% (1,274)	15.0% (417)	16.4% (224)	21.1% (2,619)
All newborns	19.6% (2,430)	46.9% (5,803)	22.5% (2,786)	11.0% (1,366)	12,385

*ASD* Atrial septal defect, *IAC* Interatrial communication, *PFO* Patent foramen ovale

^a^Fourth week of life including 255 newborns aged 28–30 days

The flow-defined PFO accounted for almost half of the PFOs (47.9%), while the channel-like PFO and the size-defined PFO were equally prevalent (26.0% and 26.1%, respectively). The most prevalent ASD subtypes were the size-defined ASD and the fenestrated ASD with equal prevalence of 2.9%, while the location-defined ASD had a prevalence of 0.2%. The median [IQR] diameters of the size-defined PFO and size-defined ASD were 2 [1; 2] mm and 4 [4; 5] mm, respectively. Of the 356 newborns with a size-defined ASD, 316 (88.8%) had a defect between 4–5 mm in diameter, and 40 (11.2%) had a defect measuring more than 5 mm in diameter. The largest size-defined ASD detected in the study was 10.5 mm. A single neonate was detected with a primum ASD (0.008% of the study population), in which a coexisting IAC in the oval fossa (size-defined ASD, 4 mm in diameter) was also found. No other types of ASDs (i.e., sinus venosus ASD or coronary sinus ASD) were detected in the study population.

### Newborn and Maternal Demographic and Echocardiographic Characteristics

Newborns found to have a PFO were slightly older at time of examination compared to newborns with an ASD or without a visible IAC (median age 12 days vs. 10 days and 10 days, respectively, *P* < 0.001). A larger proportion of newborns with an ASD were female compared to the groups of newborns with PFO and newborns without a visible IAC (53.1% vs. 46.9% and 51.0%, respectively, *P* < 1·10^–4^). A concomitant ventricular septal defect (VSD) was more common in newborns with an ASD compared to the newborns with PFO or without a visible IAC (5.6% vs. 3.3% and 2.1%, respectively, *P* = 0.001) (Table 1). In 31 (75.6%) of the newborns with ASD and a concomitant VSD, the VSD was of the muscular type, while 6 (14.6%) had a concomitant perimembraneous VSD, 3 (7.3%) had a concomitant VSD of the outlet type, and 1 (2.5%) had a concomitant VSD of the inlet type.

### Age-Specific Prevalence of IACs

**Fig. 5:**
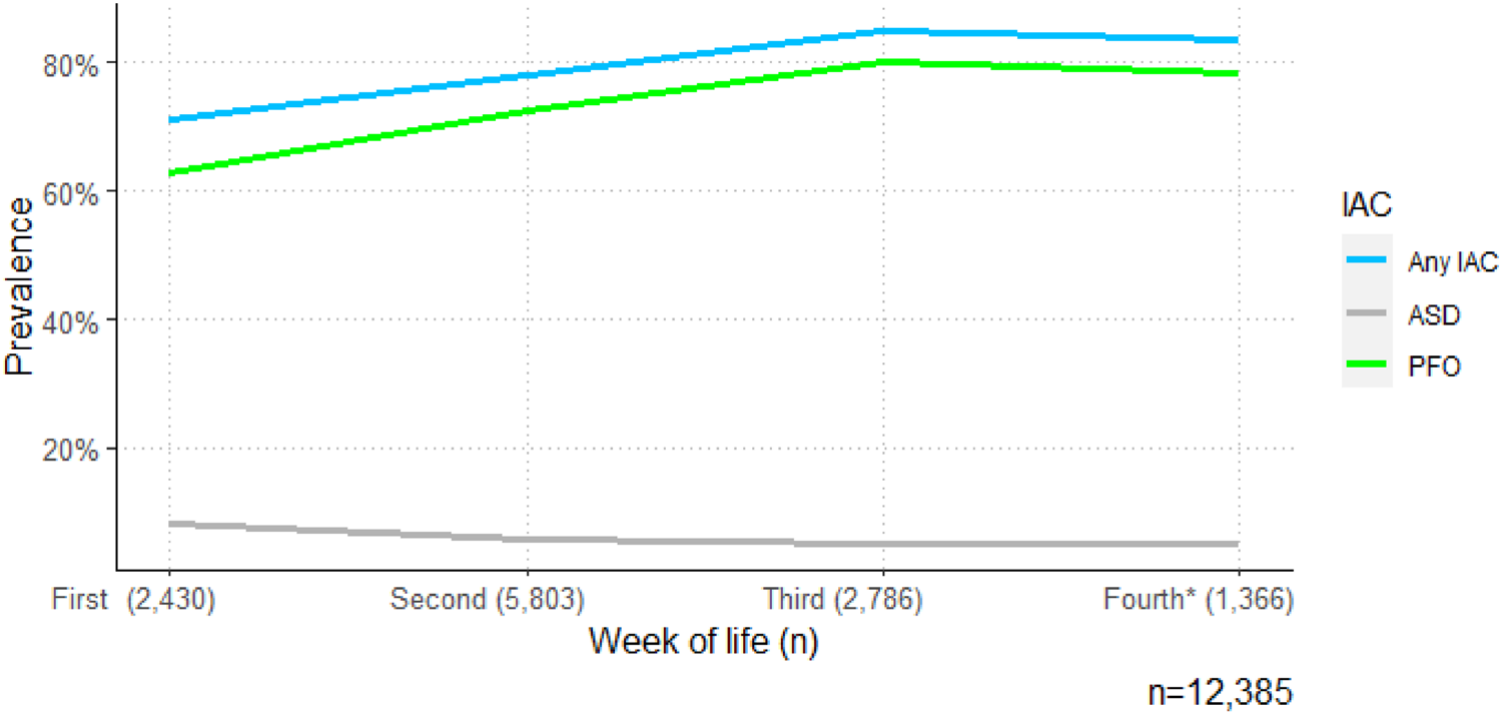
Prevalence of interatrial communications distributed into the first four weeks of life. *IAC* Interatrial communication, *ASD* Atrial septal defect, *PFO* Patent foramen ovale. *Fourth week of life including 255 newborns aged 28–30 days

When newborns were examined in their third and fourth week of life, an IAC was more often detected, compared to when newborns were examined in their first 2 weeks of life (Fig. 5).

The prevalence of ASDs was highest (8.1%) during the first week of life, decreasing to a prevalence of 5.2% in the fourth week of life. In the first week of life the most prevalent subtype of ASD was the size-defined ASD (5.3%), while in the fourth week of life the fenestrated ASD was the most prevalent (3.7%) of the ASD subtypes (Table 2).

## Discussion

In this large population study, echocardiograms from 12,385 newborns were examined for IACs using a systematic algorithm. The vast majority (78.9%) of newborns had a visible IAC distributed into PFO (72.9%) and ASD (6.0%).

The prevalence of IACs visible on TTE in newborns has previously been assessed in several studies (*n* = 36–1389) and reported to be between 24 and 92% [1–7]. Our study encompasses a much larger cohort and may more accurately estimate prevalences. In addition, in contrast to the present study, previous cohorts often consisted of selected newborns included in relation to doctor’s visits, hospitalization for other reasons [2, 3], or due to maternal illnesses such as diabetes mellitus [4, 6]. Importantly, the lack of a standardized systematic classification method of IACs has challenged the comparison of previous studies and cohorts. A uniform and unambiguous classification of IACs has previously been requested by experts [21] as this is pivotal to be able to describe the spectrum of IACs and to further investigate over time what can be considered normal and what represents pathology. One previous echocardiographic study (*n* = 1,072) did report results similar to ours with a prevalence of “interatrial septal openings” of 78.6% in newborns aged 24–72 h [5], though without differentiating between PFO and ASD. Another study reported a prevalence of visible PFO of 62% in a small cohort of selected newborns (*n* = 102, age < 57 h) with several exclusion criteria [8]. A PFO was defined by color Doppler flow through a shunt in the atrial septum with a diameter of < 3 mm while the prevalence of ASD was not reported. The prevalence was slightly lower than the prevalence of PFO found in our study. However, the definition of a PFO by Connuck et al. with a diameter of < 3 mm was lower than in the presently applied algorithm defining PFO as a diameter of < 4 mm. The threshold of a diameter of 4 mm corresponded to the 95th percentile of diameters of size-defined IACs (i.e., size-defined PFO and size-defined ASDs combined) from the original algorithm study [16], which also matches the 95th percentile of the diameter of IACs in this study of 4 mm (median [IQR] 2 [1; 2] mm). The vast majority (88.8%) of the size-defined ASDs detected in this study had a defect between 4 and 5 mm in diameter, leaving 11.2% of the size-defined ASDs with a diameter of more than 5 mm.

The prevalence of ASD in newborns in the present study was 6.0% which is higher than previous registry studies and meta-analyses of large cohorts (*n* = 7254–130,758,851) reporting prevalences of ASD between 0.05% and 1.1% [22–27] in newborns and children (age ≤ 6 years). When comparing these studies, differences in cohorts and design must be kept in mind, including more selected cohorts [24], a lack of definition of ASD [22, 25–27], or a solely size-limited definition of ASD [28]. In addition, none of these studies described how multiple fenestrations of the atrial septum were classified. In our study, the classification of IACs included a more comprehensive use of echocardiographic findings, which may have contributed to the higher prevalence of ASD than previously reported. The more detailed phenotypes described in this study are expected to contribute to the understanding of which morphological variants of the atrial septum are normal variants or abnormal and potentially pathological. This is of importance for the assessment of the natural history as well as heredity and genetic disposition to IACs. In a recently published case–control study from the CBHS based on the same study population [29], the influence of secundum ASDs (all types) was investigated on selected echocardiographic parameters (*n* = 716 cases and controls). A slight positive association between diameter size of ASD and right atrial end-systolic volume (RAESV) as well as tricuspid annular plane systolic excursion was found. Overall neonates with ASD had larger atrial volumes and larger right ventricular dimensions than matched controls, indicating that ASDs influence the morphology of the heart as early as in the neonatal period. A follow-up study of this cohort would make it possible to further determine the natural history of the ASDs examined as well as their potential influence of the cardiac morphology.

### Subtypes of IACs

The flow-defined PFO was detected by color Doppler only and represented the most common visible IAC subtype with a prevalence of 34.9%. This confirms the importance of color Doppler flow mapping in assessing IACs which is also recommended by the American Society of Echocardiography [28]. The relative difference in methods of detecting IACs, i.e., by cross-sectional visualization or by color Doppler mapping, has not been examined in previous studies and has furthermore not been applied in a large population-based cohort of newborns.

Studies investigating the prevalence of fenestrated ASDs (a.k.a. ‘multiple ASDs’) are sparse, but the management has received more focus in recent years [30]. Previous studies (*n* = 50–190) evaluating transcatheter and/or surgical closure of selected ASDs in very selected groups of patients (children and adults) reported the frequency of multiple fenestrations of the atrial septum between 9.5–30% in the included cohorts [31–33]. We found a fenestrated ASD in 2.9% of unselected newborns aged 0–30 days, and this subtype of ASD was as prevalent as the size-defined ASD, according to the new diagnostic algorithm. IACs have previously been divided into several subgroups according to diameter size with the smallest defined diameter cut-off size of 3 mm, without differentiating between PFO and ASD [5, 6]. Or, the threshold between PFO and ASD was set at 3 mm (*n* = 114) [3] or 5 mm (*n* = 90,796) [23]. In most other large cohort studies, the criteria for the ASD diagnosis were not defined [27, 34–36]. We recognize that the prevalence of the size-defined PFO and size-defined ASD depend on the cut off value of defect size.

Overall, the presently applied more detailed diagnostic criteria for ASD and PFO and their subtypes are of interest to differentiate between non-pathological versus pathological IACs, i.e., which patients are at risk of complications to the IAC, and which are not. Thus, long-term follow-up of the present cohort examined early in life is needed to answer these questions.

### Association of Demographic and Echocardiographic Findings and IACs

The observed female predominance of ASD is consistent with previous findings [24, 37–42]. In accordance with previous observations [43, 44] no relationship between maternal smoking and occurrence of PFO or ASD was found in this study. A previous study reported lower birth weight in 471 newborns with ASD compared to a control group without ASD [41], resembling the findings in our study. More newborns with an ASD visible on TTE also had a VSD (5.6%) compared to newborns with PFO (3.3%) and newborns without an IAC (2.9%) (*P* = 0.001). The prevalence of VSD in unselected newborns (*n* = 25,556) have previously been reported by the CBHS to be 3.3%, of which almost 9/10 closed spontaneously within the first year after birth [45].

### Age-Specific Prevalence of Subtypes of IACs

The prevalence of a flow-defined PFO or channel-like PFO increased with age during the first 30 days of life, while a size-defined PFO decreased in prevalence. Size-defined ASDs were more prevalent in the first week of life, as opposed to more fenestrated ASDs in the fourth week of life. This change in subtypes could be explained by the hemodynamical transitional changes in the neonatal circulation during the first month after birth including closing of the ductus arteriosus and expansion and ventilation of the lungs [46–49]. With the pressure gradient over the atrial septum changing to left-to-right [50] lowering the right atrial pressure, the relative right atrial volume possibly decreases. Following this, an IAC presenting as an open defect in the interatrial septum (like the size-defined PFO) might change its morphology to either narrow down to form a channel-like structure or collapse so that blood only is permitted through the communication during periods in the cardiac cycle with a high interatrial pressure gradient. Consequently, a size-defined PFO in a few days old newborn might change configuration to either a channel-like or flow-defined PFO later on. The rise in left-to-right pressure gradient could also explain the increase in prevalence of fenestrated ASDs, as increased blood flow through the fenestrations may increase their detectability with Doppler. More IACs were detected in newborns aged 14–30 days compared to those aged 0–13 days. Previous studies have shown an immediate and continuous fall in right atrial and pulmonary arterial pressures within at least the first 14 days postpartum [50–55]. Despite the lack of studies performing repeated measurements of right atrial or pulmonary arterial pressures or volume in newborns during the first month of life, the possible change in pressure gradient could explain the change in subtypes of IACs observed in our study. Consequently, hemodynamic changes in the neonatal period could explain the frequency of IACs seen at different timepoints after delivery.

### Strengths and Limitations

The considerable strengths of our study are the size of the cohort, the population-based study design, and the systematic approach to analysis of the TTE images. The newborns included in our study was examined with a standardized pediatric echocardiogram as part of a population-based cohort study rather than detected with an ASD due to symptoms. Despite the population-based design of Copenhagen Baby Heart Study, not all children born in the study period were included which may introduce selection bias [18]. Newborns may already have been diagnosed prenatally or shortly after birth and entered routine clinical management, and, although encouraged to participate, might not have been included in this study thus lowering the prevalence of the more serious types of IACs. In addition, participants were predominantly Caucasian, suggesting that our results might not be generalizable to other ethnicities [18]. The echocardiographic analyses were performed by two sonographers trained in pediatric echocardiography, simultaneously leading to consistency in analysis but also to risk of systematic bias. As each newborn included in this study was examined only once, the evaluation of the change in age-specific prevalence of the subtypes during the first month of life is not longitudinal.

### Clinical Impact

This study demonstrates that a systematic classification of IACs can be used in a large cohort of newborns and provides clinicians with a useful tool to identify infants with visible PFO and ASD on TTE. The new diagnostic algorithm gives clinicians a standardized systematic classification method to detect, classify, and diagnose IACs at bedside, thereby also unifying the diagnosis among clinicians and aid genetic analysis. It is yet unknown which subtypes of IACs lead to complications later in life, and in which cases early intervention can prevent these. A recent study found that 89% of term newborns with a secundum ASD of ≥ 3 mm experienced spontaneous closure or diminution of size to < 3 mm [56]. Distinguishing which IACs require follow-up and intervention would be useful and could furthermore help in understanding heredity and genetic disposition and will depend on long-term findings.

## Conclusion

In a large population-based cohort of 12,385 newborns, 78.9% had a visible IAC, which was classified as PFO in 72.9% and ASD in 6.0%. The most frequently visible subtype of PFO was the size-defined PFO. The equally most prevalent subtypes of ASD were the size-defined ASD and the fenestrated ASD. The study demonstrated that a systematic classification of IACs could provide clinicians with a useful tool to identify infants with PFOs and ASDs visible on transthoracic echocardiography.

## Supplementary Information

Below is the link to the electronic supplementary material.

Supplementary file1 (DOCX 1025 kb)

## Data Availability

The CBHS welcomes proposals for collaboration and encourages interested parties to contact us at [hgh-babyheart@regionh.dk]. Per Danish and EU data protection law, CBHS data cannot be uploaded to publicly accessible data repositories. However, data can be made available to specific, well-defined projects upon receipt of approval from the Danish Data Protection Agency, the Scientific Ethics Committees of the Capital City Region of Denmark, and the CBHS’ steering committee.

## Abbreviations

ASD: Atrial septal defect
IAC: Interatrial communication
PFO: Patent foramen ovale
TTE: Transthoracic echocardiography
VSD: Ventricular septal defect
