# Supplementary material for: Interatrial Communications: Prevalence and Subtypes in 12,385 Newborns–a Copenhagen Baby Heart Study
Source: Pediatr Cardiol. 2024 Jul 13;46(6):1570–80. doi: 10.1007/s00246-024-03571-0 (PMC12296769; doi:10.1007/s00246-024-03571-0)
Supplement: Supplementary file 1 — Supplementary file1 (DOCX 1025 kb) [file 246_2024_3571_MOESM1_ESM.docx]

**SUPPLEMENTARY APPENDIX: Associated description of algorithm, and the Copenhagen Baby Heart Study transthoracic echocardiographic protocol**

Supplementary figures 1-2 from Dannesbo, S. et al., 2022: *A novel algorithm for classification of interatrial communications within the oval fossa in the newborn - a Copenhagen Baby Heart substudy.* *Cardio Young.*

**Supplementary figure 1 -** Image examples of the subtypes.
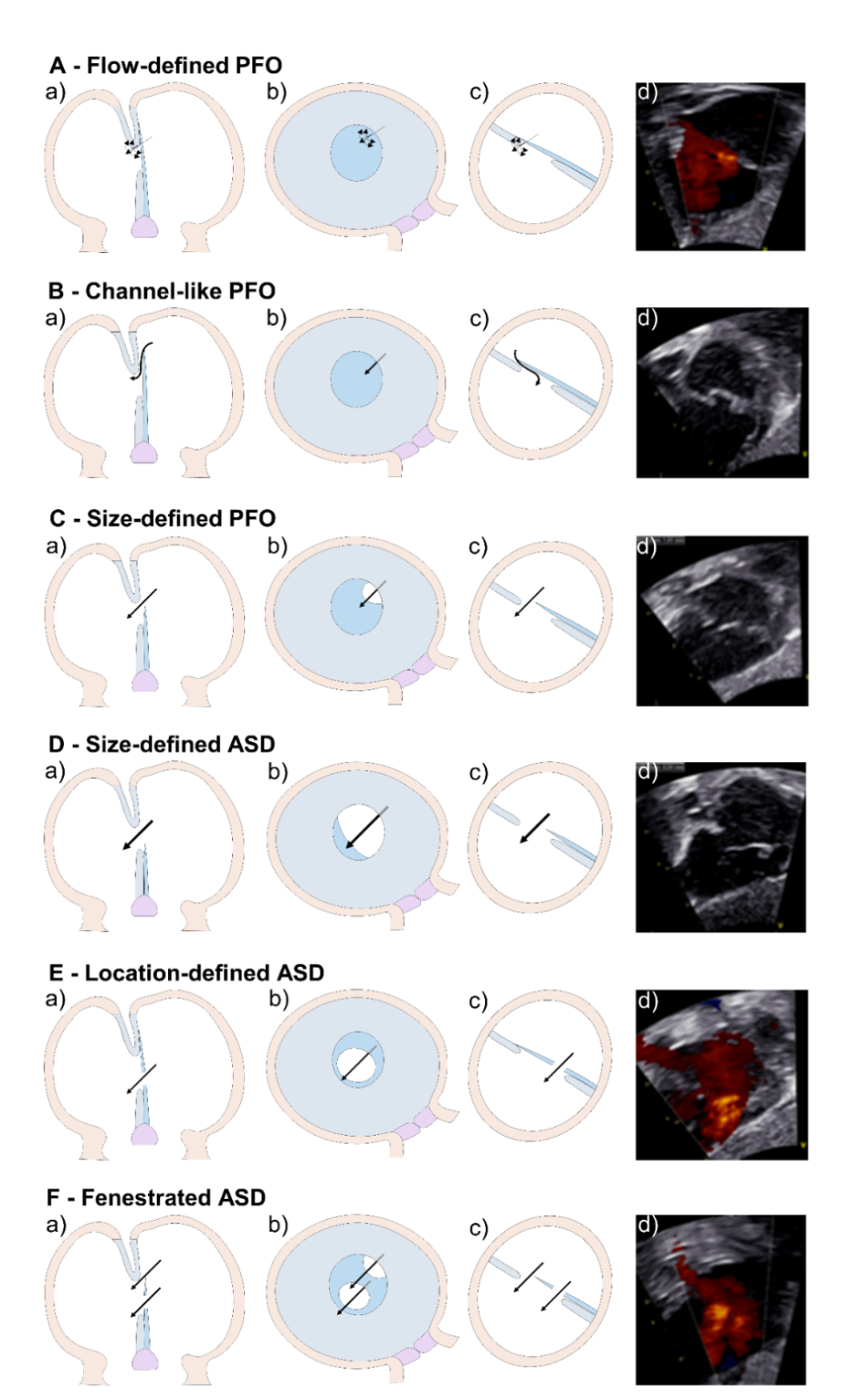


**Supplementary figure 2 -** Pitfalls to be aware of (see image below)

- To be able to detect an interatrial communication following the algorithm, one needs images of acceptable quality in the correct projection without too much noise.
- If aorta is visualized by its blue color flow across the atria, the projection is not acceptable. (Image 1a and 1b)
- Dropouts of the ultrasound signal from the cardiac tissue can occur, which can be mistaken for visual communications in the septum. To ensure a communication is not a dropout there needs to be consistency between point of color flow and visual communication. (Image 2a and 2b)
- If the color flow is perpendicular to the axis of the probe, the signal from the flow of blood can be weakened. The same phenomenon results in dropouts of the signal from the septum, if the septum is parallel to the axis of the probe.
- In the case of a large color jet from the inflow of superior vena cava one should be aware of not mistaken bleeding from the superior vena cava flow with color flow crossing the atrial septum. This can be evaluated by turning down color Doppler gain. (Image 3)
- Turbulence of blood flow from inferior vena cava against the inferior part of the septal wall can be mistaken for flow acceleration across the septum. Eustatic valves and Chiari network can be the cause of turbulence in this area as well. (Image 4)
- In the case of multiple flows crossing the septum one needs to ensure that each flow meets the criteria of either a visible communication or a non-visible communication with flow acceleration in the color flow cf. the algorithm. A visible communication can occur together with a non-visible communication with flow acceleration.
- If a convincing visible communication is visualized in one cycle, but not in the rest of the loop, this finding will overrule (a non-visible communication).


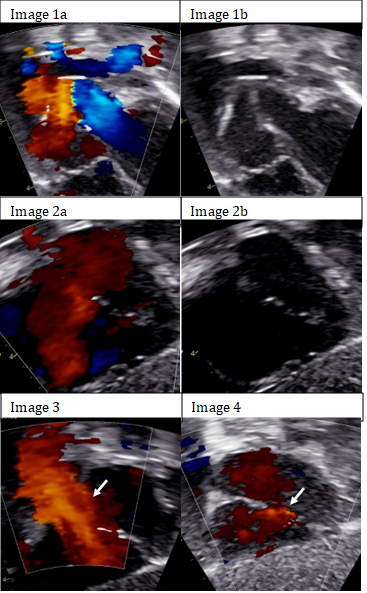


Corresponding author of supplementary figures: Sofie Dannesbo, MD, Department of Cardiology, Copenhagen University Hospital Herlev, Department of Cardiology, The Heart Centre, Copenhagen University Hospital Rigshospitalet, and Department of Clinical Medicine, University of Copenhagen, Copenhagen, Denmark.

Email: [sofie.dannesbo.01@regionh.dk](mailto:sofie.dannesbo.01@regionh.dk)
